# Association of Preexisting Drug-Resistance Mutations and Treatment Failure in Hepatitis B Patients

**DOI:** 10.1371/journal.pone.0067606

**Published:** 2013-07-30

**Authors:** Jie Ma, Yingchun Zhang, Xinyue Chen, Yi Jin, Dexi Chen, Yun Wu, Jing Cui, Haitao Wang, Jia Liu, Ning Li, Feng Gao

**Affiliations:** 1 Beijing Institute of Liver Disease, Beijing, China; 2 Beijing Baihuirui Bio-Technologies Inc, Beijing, China; 3 Department of Medicine, Beijing You'an Hospital, Capital Medical University, Beijing, China; 4 Department of Surgery, Beijing You'an Hospital, Capital Medical University, Beijing, China; 5 Department of Microbiology, Peking University Health Science Center, Beijing, China; 6 Department of Medicine, Duke University Medical Center, Durham, North Carolina, United States of America; Saint Louis University, United States of America

## Abstract

The role of preexisting minority drug-resistance mutations in treatment failure has not been fully understood in chronic hepatitis B patients. To understand mechanisms of drug resistance, we analyzed drug-resistance mutations in 46 treatment-failure patients and in 29 treatment-naïve patients and determined linkage patterns of the drug-resistance mutations in individual viral genomes using a highly sensitive parallel allele-specific sequencing (PASS) method. Lamivudine resistance (LAMr) mutations were predominant in treatment-failure patients, irrespective of the inclusion of LAM in the regimen. The primary LAMr mutations M204V and M204I were detected in 100% and 30% of the treatment-failure patients, respectively. Two secondary LAMr mutations (L180M and V173L) were also found in most treatment-failure patients (87% and 78%, respectively). The linkages containing these three mutations dominated the resistant viruses. Importantly, minority LAMr mutations present in <2% of the viral population were detected in 83% of the treatment-naïve patients. Moreover, the low-frequency same linked LAMr mutations (<0.15%) were detected in 24% of the treatment-naïve patients. Our results demonstrate that the selection of preexisting minority linked LAMr mutations may be an important mechanism for the rapid development of LAM resistance, caution the continuous use of LAM to treat drug-experienced and -naïve hepatitis B patients, and underline the importance of the detection of minority single and linked drug-resistance mutations before initiating antiviral therapy.

## Introduction

Hepatitis B virus (HBV) infection affects ∼350 million people worldwide [1], and 15%–40% of chronic hepatitis B (CHB) patients can develop cirrhosis, hepatic failure, and hepatocellular carcinoma in their lifetime [2]. However, disease progression and quality of life have been significantly improved when patients were treated with interferon and nucleoside/nucleotide analogs (NAs) [3]. NAs are currently considered a primary therapeutic option for CHB patients, and the initial response to this therapy is favorable in the majority of patients. However, the limitation of long-term NA treatment is the emergence of drug-resistance mutations, followed by virological breakthrough and hepatitis flare [4]–[6].

Among five NAs, lamivudine (LAM) was the first NA to be approved for the treatment of HBV patients [6], [7]. Patients treated with LAM quickly develop resistance due to the emergence of primary M204V and M204I mutations, which can significantly affect viral fitness [8]–[11]. Both primary mutations are often accompanied by secondary/compensatory mutations (L180M/I, V173L, and L80V/I), which can either increase the level of the resistance or restore the fitness loss [12]–[16]. The genetic barrier to LAM resistance (LAMr) is considered to be the lowest among all NAs. However, the mechanisms of such a low genetic barrier have not been fully elucidated. Because multiple mutations in the same viral genome are required for viruses to become more resistant to LAM and other NAs, determining individual and linked drug-resistance mutations in treatment-failure and -naïve patients is critical to understand the mechanisms of resistance to NAs. We have recently developed a highly sensitive parallel allele-specific sequencing (PASS) method to detect minority drug-resistance mutations (<0.01%) and determine linkage patterns of multiple drug-resistance mutations in HIV-1-infected individuals [17]–[19]. Here, we analyzed the HBV drug-resistance mutations in 46 treatment-failure patients and 29 treatment-naïve patients using the PASS assay to study drug-resistance mechanisms in CHB patients.

## Materials and Methods

### Study population

Patients who visited You'an Hospital in Beijing, China were selected from the HBV Patient Sample Repository database after a review of the characteristics of their treatment history. All patients were treated based on the treatment guideline established in China [20]. Prior treatment history was obtained through hospital records or questionnaire for the use of prescribed and non-prescribed drugs. The written consent was obtained from all patients who participated in the study, and the study was approved by the ethics committee of You'an Hospital. Consecutive plasma samples from each patient were collected from 2009–2012 for routine clinical lab assays, and one residual plasma sample after treatment failure from each patient was used to analyze drug-resistant mutations. The treatment-failure patients who had likely developed drug resistance were included for this study: 21 patients who developed virological resistance (a viral load [VL] increase >1.0 log_10_ IU/mL in two consecutive serum samples taken one month apart), and 25 patients in whom VLs were not decreased >1.0 log_10_ IU/mL but persisted at levels >3 log_10_ IU/mL during antiviral therapy. The average VL in the treatment-failure patients was 5.2 (3.0–7.9) log_10_ IU/mL. The patients were treated with NAs (individually or in combination) in a single regimen or in multiple consecutive regimens with LAM, telbivudine (LdT), entecavir (ETV), and adefovir (ADV). Among 46 treatment-failure patients, 27 were treated with regimens that contained LAM and 19 were not treated with LAM regimens. Twenty-nine CHB patients were not treated with NAs. The average VL was 7.2 (5.5–8.0) log_10_ IU/mL.

### Detection of drug-resistance mutations by PASS

The PASS assay was performed to amplify partial reverse transcriptase (RT) gene (1,017 bp) as previously described [17], [18]. Briefly, 20 µl of 6% acrylamide gel mix, containing 1 µM acrydite-modified reverse primer BRTR3 5′Acr-GAGCCACAAAGGTTCCACGCAT-3′ (nt1241–1263), HBV DNA template, 0.3% diallyltartramide, 5% rhinohide polyacrylamide gel strengthener (Molecular Probes, Eugene, OR), 0.1% ammonium persulfate (APS), 0.1% TEMED (*N,N,N′,N′*-tetramethylethylenediamine), and 0.2% bovine serum albumin (BSA), was used to cast a gel on a bind-silane (Amersham Biosciences, Piscataway, NJ) treated glass slide. Various amounts of HBV DNA (5 µl to 18.5 µl) were used for the PASS assay to obtain an optimal number of viral genomes (between 1,000 and 2,000, or as many as possible with low VL samples) in each assay. The in-gel PCR amplification was then performed in a PTC-200 thermal cycler with a mix of 1 µM forward primer BRT1 5′-AGTCTAGACTCGTGGTGGACTTCTCTCA-3′ (nt247–274), 0.1% Tween 20, 0.2% BSA, 1× PCR buffer, 100 µM deoxynucleoside triphosphate (dNTP) mix, 3.3 U of Jumpstart *Taq* DNA polymerase (Sigma, St. Louis, MO), and H_2_O (up to 300 µl) under a sealed SecurSeal chamber (Grace Bio-Labs, Inc., Bend, OR). The PCR conditions were as follows: 94°C for 3 min; 65 cycle of 94°C for 30 s, 56°C for 45 s, and 72°C for 3 min; 72°C for 6 min.

After in-gel PCR amplification, single-base extension (SBE) was performed using the sequencing primer that was annealed just upstream of the mutation site with mutant and wild type (wt) bases that were distinctively labeled with Cy3 and Cy5, respectively. To detect multiple drug-resistance mutations on the same viral genome, the immobilized PCR products in each gel were sequentially interrogated by 13 SBE reactions using the primers for the following mutations: L80V, L80I, V173L, L180M, A181V, A181T, T184G, A194T, S202I, M204V, M204I, N236T, and M250V [6], [21]. After each SBE, the gel was scanned with a GenePix 4200B Microarray Scanner (Molecular Devices, Sunnyvale, CA) to acquire images. The two channel images (Cy3 for wt and Cy5 for mutant) that were acquired from each PASS assay were analyzed with Progenesis PG200 software (Nonlinear Dynamics, Durham, NC). After background subtraction and normalization, only the unambiguous spots at both channels were included for further analysis. The normalized pixel count data at multiple mutation sites for each spot were exported into an Excel file with a unique number. By comparing the normalized values of each spot at two channels, the position was classified as wt or mutant. Finally, the linkage pattern of all of the mutations on each viral genome was determined using the Linksys program as previously described [17], [18].

### Detection of drug-resistance mutations by sequencing

The partial RT gene was amplified using the extracted HBV DNA and the PCR products were directly sequenced as described [22]. The drug-resistance mutations were determined using the HBV-Resistance Interpretation Tool Algorithm, version 03-2007 (http://www.hiv-grade.de/hbv_grade/deployed/grade.pl?program=hbvalg&action=showSequenceForm). The sequences were aligned with genotype reference sequences using CLUSTAL W. A neighbor-joining phylogenetic tree using the Kimura two-parameter model was constructed to determine genotypes. The GenBank accession numbers for the sequences generated in this study are KC907128-KC907202.

### Statistical Analysis

The statistical analysis was performed using SPSS software, version 16.0. The categorized variable data were analyzed using Fisher's exact, Continuity Correction, Pearson Chi-Square tests. The continuous variable data were analyzed using the Student's *t* test when the variables were normally distributed by the non-parametric Kolmogorov-Smirnov test, or using the non-parametric Mann-Whitney *U* test when the variables were not normally distributed. A *p* value of less than 0.05 was considered statistically significant.

## Results

### LAMr mutations were detected in all treatment-failure patients

**Figure 1:**
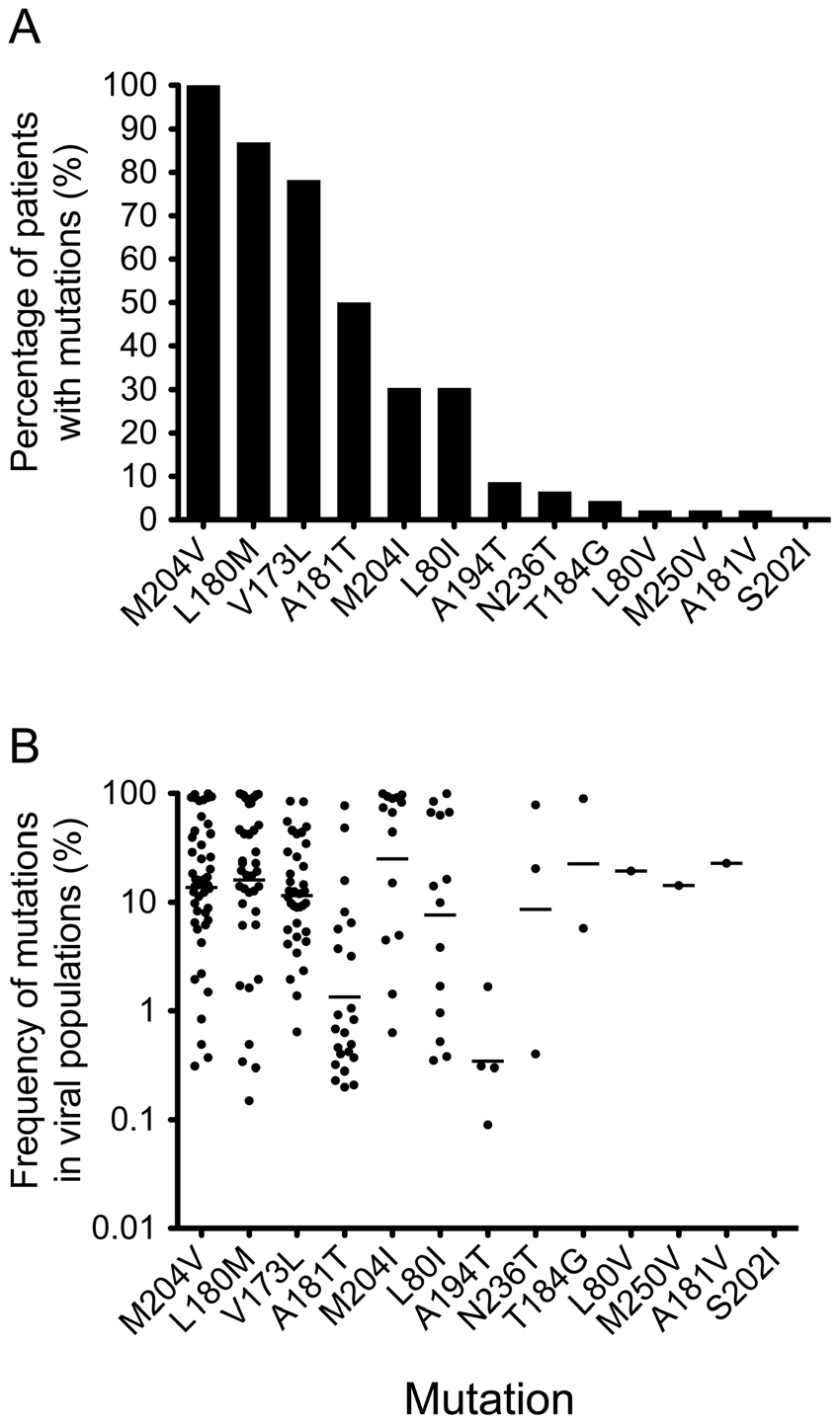
Detection of drug-resistance mutations in treatment-failure patients. HBV genomic DNA was extracted from the plasma samples of treatment-failure patients, and 13 primary and secondary/compensatory drug-resistance mutations were analyzed by PASS. The percentages of patients with individual drug-resistance mutations (A) and the frequencies of each mutation in the viral population (B) were determined. Each dot indicates the frequency of a mutation in the viral population in a patient. The geometric means of the mutation frequencies in the viral populations are shown as bars.

The primary drug-resistance mutations (A181V, A181T, T184G, A194T, S202I, N236T, M204V, M204I, and M250V) and secondary/compensatory mutations (L80V, L80I, L180M, and V173L) [6], [21] associated with four NAs (LAM, ADV, ETV, and LdT) that were used to treat patients were analyzed using the highly sensitive PASS assay. An average of 827 (76–1,701) viral genomes from each patient were analyzed, and an average of 4 (2–7) drug-resistance mutations were detected in each patient (Table 1). Six predominant drug-resistance mutations (M204V, L180M, V173L, A181T, M204I and L80I) were detected in 30%–100% of the patients and they were all associated with LAM resistance, whereas the other seven mutations (A194T, N236T, T184G, L80V, M250V, A181V and S202I) were detected in <10% of the patients (Fig. 1A). The percentages of all six mutations detected in 30%–100% of the patients were significantly higher than those of seven mutations detected in <10% of the patients (*p*<0.02). The primary LAMr mutation M204V was detected in all 46 treatment-failure patients, while the primary LAMr mutation M204I was found in 30% of the patients. Two secondary/compensatory LAMr mutations, L180M and V173L, were also detected in most patients (87% and 78%, respectively). The secondary/compensatory LAMr mutation L80I was present in relatively fewer patients (30%). The A181T mutation, which is cross-resistant among LAM, ADV, and LdT [6], [21], was frequently detected (50%). The mean frequencies of four LAMr-associated mutations (M204V, M204I, L180M, and V173I) were >10% (11.5%–25.2%) of the viral populations (Fig. 1B). The A181T mutation was present at a much lower frequency (1.3%).

**Table 1:** Detection of drug-resistance mutations in treatment failure patients by PASS.

Patient	Genotype	Treatment (months on therapy)	VL (Log_10_ IU/ml)	Increase of VL from nadir (Log10 IU/ml)	HBeAg	No. of analyzed genomes	% of viruses with mutations	No. of mutations	No. of linkages
N001	B	ADV(9)	4.3	0.8*	+	1,180	1.95	4	2
N002	C	ADV(12)	5.4	0.7*	+	752	37.5	3	1
N003	C	LAM(4)→ADV(4)→ETV(23)	5.5	2.8	−	628	97.77	3	2
N004	C	ETV(3)	4.2	0.5*	+	320	49.37	4	2
N005	C	LdT(24)	4.5	1.5	+	203	99.01	2	2
N006	C	ADV(50)	4.9	1.2	+	150	19.33	4	2
N007	C	ADV(7)	5.8	0.8*	+	1,360	6.18	3	1
N008	B	LAM(14)	7.6	0.0*	+	1,596	99.69	3	2
N009	C	ADV(24)	5.1	0.1*	+	1,625	3.52	6	2
N010	C	ADV(35)	3.6	0.9*	+	161	49.69	5	3
N011	C	LAM(9)	3.0	0.3*	+	649	14.95	3	2
N012	C	ADV(4)	4.3	1.0*	+	788	65.99	4	4
N013	C	ADV(25)	5.9	3.2	+	2,013	18.53	5	2
N014	C	LdT(18)	6.6	3.9	+	1,556	99.61	3	3
N015	C	LAM(7)	5.3	0.9*	+	2,495	16.83	2	4
N016	C	ADV(20)	5.6	1.4	+	1,457	9.27	3	3
N017	B	LdT(6)	4.6	1.9	+	146	8.22	7	2
N018	C	LdT(16)→LdT+LAM(7)→LdT(1)	4.5	1.8	+	1,399	89.78	4	1
N019	C	LAM(24)→ADV(31)	5.9	1.9	−	526	82.7	4	3
N020	C	ADV(22)→ADV+LAM(12)→ETV(1)	5.4	2.4	+	1,338	73.32	4	3
N021	C	LAM(48)→ADV(2)→ADV+LAM(3)→ADV(19)	7.9	4.5	+	812	68.8	4	3
N022	B	LAM(7)→ADV(4)	5.4	1.1	+	654	1.07	4	1
N023	C	LdT(3)→ADV(6)	5.9	3.2	+	962	8.73	3	1
N024	C	ETV(35)→ADV(4)	7.7	2.5	+	848	5.07	3	1
N025	B	LdT(3)→ADV(7)→LdT(7)→ADV(7)	7.0	3.0	+	374	15.51	3	1
N026	C	ADV(3)→LAM(25)	3.3	0.6*	+	1,323	29.63	6	2
N027	C	ADV(16)→LAM(2)→LAM+ADV(11)	4.4	0.0*	+	217	92.16	6	8
N028	C	ADV(5)→LdT(21)	3.4	0.7*	−	350	98.57	3	0
N029	C	ADV(14)→ADV+LdT(1)→LdT(17)	6.2	1.9	+	313	82.11	3	8
N030	B	LAM(13)→LAM+ADV(1)→ADV(4)	4.9	0.1*	+	1,131	13.97	4	4
N031	C	ADV(12)→LAM(19)	7.6	4.9	+	311	100	4	5
N032	B	LAM(36)→ADV(12)→ADV(43)	3.8	1.1	+	321	21.5	2	2
N033	C	ADV(7)→ETV(6)	3.2	0.3*	+	371	100	6	4
N034	B	ADV(3)→LAM+ADV(17)	6.3	2.7	+	707	14.43	7	3
N035	B	ETV(31)→LAM(10)	3.2	0.5*	+	468	34.4	5	5
N036	C	LAM(6)→ADV(83)	6.7	0.0*	+	785	89.68	4	2
N037	C	LAM(16)→ADV(24)	6.2	1.1	+	768	63.28	6	3
N038	C	LAM(1)→LAM(9)→ETV(3)→LAM+ADV(3)	5.0	1.0*	+	1,641	42.9	4	1
N039	C	ADV(8)→LdT(14)→LAM+ADV(6)	4.0	0.5*	−	744	98.12	4	2
N040	C	LAM(7)→ADV(17)→ADV(1)	5.2	2.5	+	239	18.83	5	4
N041	C	LAM+ADV(18)→None(8)→LAM(12)	7.8	4.7	+	497	96.18	3	4
N042	C	LAM(1)→ADV(18)→None(3)→LdT(13)→None(6)→LdT(18)	7.2	4.5	+	472	100	4	4
N043	C	LAM(60)→ADV(30)→ETV(13)	4.2	1.5	+	211	99.05	4	6
N044	C	LAM(14)→None(2)→LAM(3)→LAM+ETV(14)→ETV+ADV(26)	3.5	0.0*	+	1,710	99.71	6	5
N045	D	LAM(24)→LAM+ADV(4)→ADV+LdT(5)	3.6	0.9*	−	76	90.79	3	2
N046	C	LdT(5)→LAM+ADV(4)→ADV(4)	4.5	1.8	+	1,407	1.92	3	1
Mean			5.2 (3–7.9)	1.6 (0–4.9)		827 (76–1,701)	31.72 (1.07–100)	4.02 (2–7)	2.8 (0–8)

Drug abbreviation: lemivudine (LAM), telbivudine (LdT), entecavir (ETV), and adefovir (ADV). None: no treatment.

Arrow: change of treatment regimens.

Asterisk: The viral load increase ≤1.0 log10 IU/mL) from nadir.

Nearly half of the patients (41%) were not treated with LAM, but none of mutations specifically associated with ETV and ADV resistance (N236T, T184G, M250V, and S202I) were detected in more than 3 patients (Fig. 1A). However, their frequencies in the viral populations were relatively high (>8%) (Fig. 1B). No differences in the percentage of drug-resistance mutations (26.04%±1.09% versus 21.45%±1.15%; *p* = 0.3) or in the frequency of mutations in the viral population (16.70%±2.40% versus 12.98%±2.16%; *p* = 0.5) were observed in patients who were treated with or without LAM. Less than 7% (1.1%–6.2%) of the viral population was found to contain drug resistance-associated mutations in six patients (Table 1). Interestingly, all of the patients were treated with ADV either individually or in combination with other drugs in the current regimen when the drug resistance-associated mutations were analyzed. Overall, there was no correlation between the level of viremia and percentage of viruses with mutations in the sample (*r* = 0.068; *p* = 0.66).

Drug resistance-associated mutations were detected in less than half (46%) of the treatment-failure patients by population sequencing (Table S1). 141 mutations detected by PASS were missed by the sequencing method, while only two mutations detected by sequencing were missed by PASS due to the variations at the primer binding sites. This demonstrated that the PASS assay was much more sensitive for detection of minority mutations than the commonly used population sequencing method.

Patients in this cohort were infected with three genotypes: B (9), C (36) and D (1) (Table 1). The frequencies of viruses with drug-resistant mutations were significantly higher in genotype B than genotype C (61.4% verses 23.6%; *p* = 0.007). The A181T mutation was significantly more in genotype C than genotype B (61% versus 11%; *p* = 0.021). We also observed higher percentage of the M204I mutation in genotype C (36.1%) than genotype B (11.1%) as previously reported [23], but the difference was not statistically significant due to the small sample size (*p* = 0.3). Three mutations (L173L, L180M and A194T) were more frequently detected in genotype B than genotype C, but none of the differences were statistically significant.

### Linked LAMr mutations predominated in treatment-failure patients

**Figure 2:**
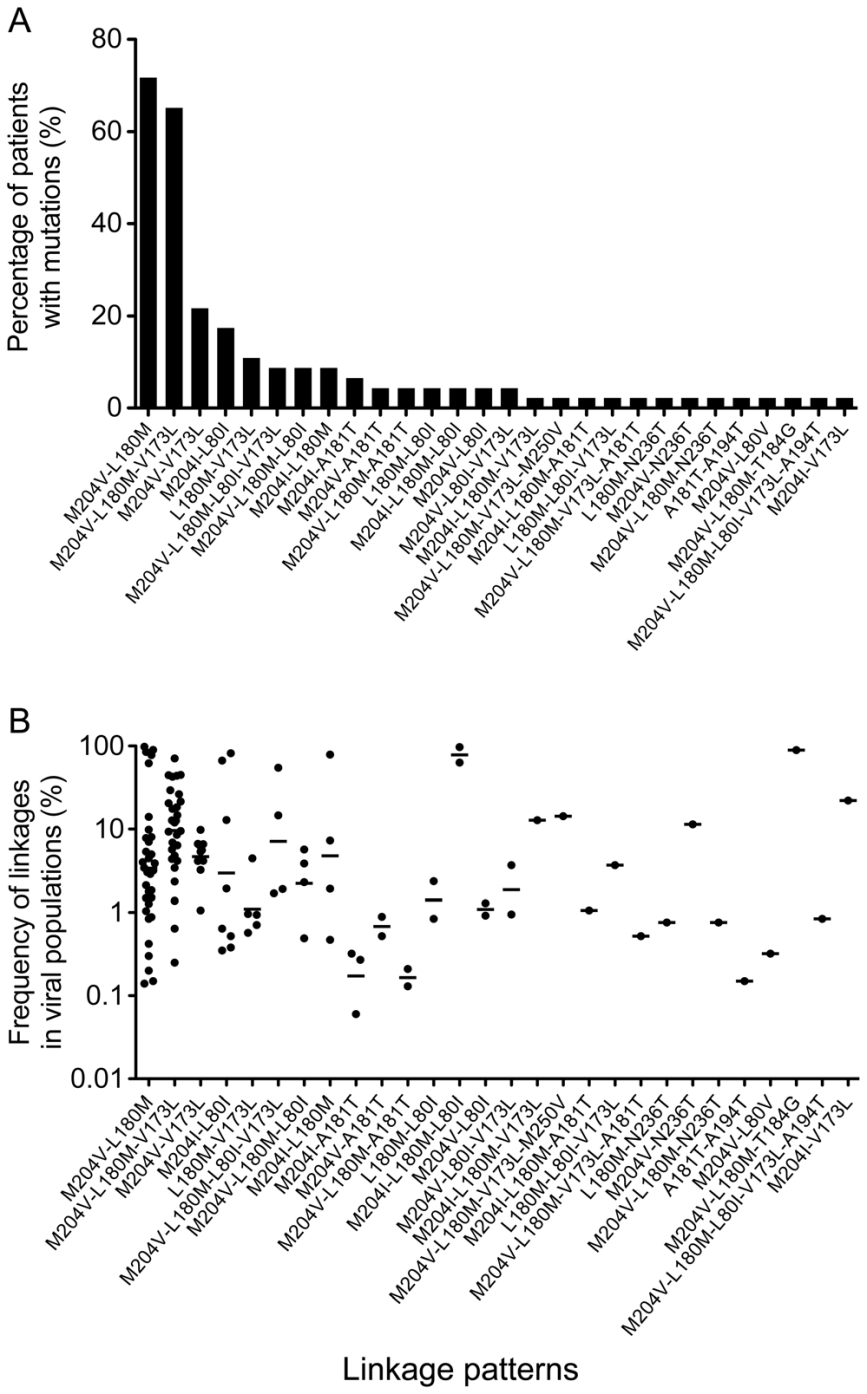
Linkage analysis of the drug-resistance mutations in individual viral genomes from treatment-failure patients. The linkage relationship among multiple drug-resistance mutations in each viral genome was analyzed using the Linksys program. The percentage of patients with different linkage patterns (A) and the frequencies of each linkage pattern in the viral population (B) were determined. Each dot indicates the frequency of a linkage of mutations in the viral population in a patient. The geometric means of the frequencies of viruses with different linkage patterns in the viral populations are shown as bars.

Although multiple drug-resistance mutations are found in individual treatment-failure patients, the linkage relationships of these mutations have only been studied with a limited number of clones from patients in a few studies [23]–[25]. Recently, the ultra-deep pyrosequencying (UDPS) method was used to characterize the linkage patterns; however, this method was limited to mutations within a short genome region [26]. Therefore, we sought to characterize the linkage relationship among the 13 mutations and its potential implication in drug resistance. Twenty-eight linkage patterns were identified (Fig. 2A) and an average of 2.8 (0–8) linkage patterns were detected in each patient (Table 1).

The most frequent eight linkage patterns exclusively contained LAMr mutations (M204V, M204I, L180M, V173L, and L80I), whereas two-thirds of other linkage patterns were only detected in 1–2 patients (Fig. 2A). The two most predominant linkages were M204V-L180M and M204V-L180M-V173L, which were detected in 71% and 65% of the treatment-failure patients, respectively. All other linkages were present in fewer than 22% of the patients. The percentages of the two predominant linkages were significantly higher than those of all the other linkages (*p*<0.001). The M204V-L180M-V173L linkage (9.6%) was present more frequently in the viral population than the M204V-L180M linkage (3.4%) (Fig. 2B). The next two linkage patterns with higher percentages were M204V-V173L and M204I-L80I (22% and 17%, respectively; Fig. 2A). The percentage of patients with the M204V-L180M linkage and the frequency of the M204V-L180M linkage in the viral population were significantly higher in patients treated with LAM than in untreated patients (*p* = 0.016 and *p* = 0.026, respectively), suggesting that this linkage was more likely selected during treatment regimens containing LAM. The majority of the patients (87%) had the M204V-L180M linkage pattern alone or with additional mutations, although nearly half of the patients (41%) were not treated with LAM. These results indicate that viruses with linked LAMr mutations were widely present in treatment-failure patients and will likely be resistant to LAM regardless of whether patients are on antiviral therapy with or without LAM.

The linkages between the non-LAMr mutations (T184G, A194T, N236T, and M250V) and the primary LAMr mutations were detected only in a few patients. However, some of these linked mutations were present at high frequencies in the viral population, for example, 89.4% for the M204V-L180M-T184G linkage (Fig. 2B). This result indicates that viruses with these linked drug-resistance mutations may potentially render the patient resistant to both nucleoside and nucleotide analogs in salvage treatment regimens [27], [28].

### Linked LAMr mutations were frequently detected in treatment-naïve patients

**Figure 3:**
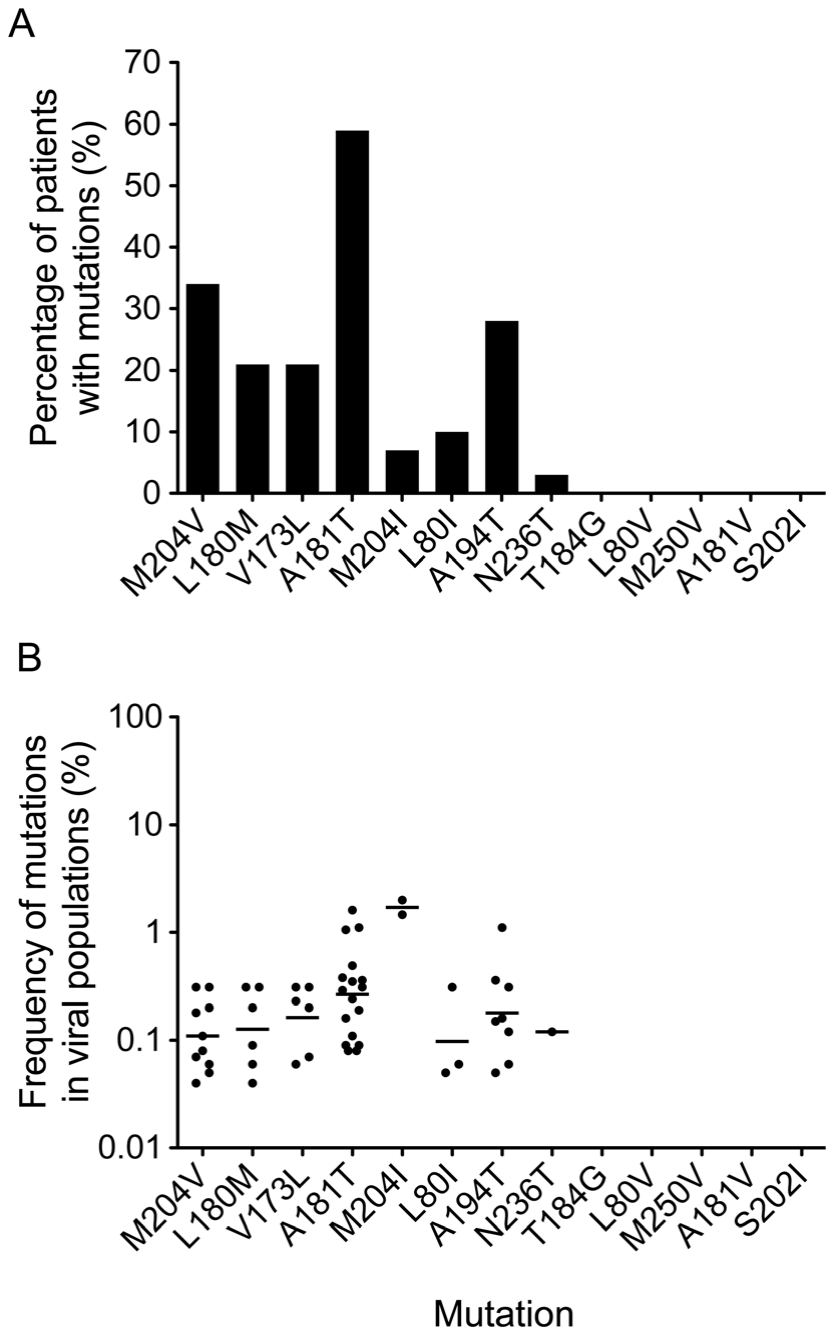
Detection of drug-resistance mutations in treatment-naïve patients. HBV genomic DNA was extracted from the plasma samples of treatment-naïve patients, and 13 primary and secondary/compensatory drug-resistance mutations were analyzed by PASS. The percentages of patients with individual drug-resistance mutations (A) and the frequencies of each mutation in the viral population (B) were determined. Each dot indicates the frequency of a mutation in the viral population in a patient. The geometric means of the mutation frequencies in the viral populations are shown as bars.

Because linked LAMr mutations were predominant in the treatment-failure patients, we next sought to investigate whether they could be detected in 29 treatment-naïve patients. Since VLs in treatment-naïve patients were significantly higher than those in treatment-failure patients (*p*<0.001), this allowed us to analyze a larger number of viral genomes in each patient and increased the likelihood to detect low-frequency drug-resistance mutations. An average of 1,257 (317–2,772) viral genomes per patient were characterized. Drug-resistance mutations were detected in most patients (83%), and an average of 1.3 (0–5) drug-resistance mutations were detected in each patient (Table 2). Similar as in the treatment-failure patients, the LAMr mutations (M204V, L180M, V173L, A181T, and L80I) were predominant in the treatment-naïve patients (Fig. 3A and Table S2). However, the percentages of the treatment-naïve patients with four LAMr mutations (M204V, L180M, V173L, and M204I) were significantly lower than those of the treatment-failure patients (*p*<0.001). Moreover, the mean frequencies of the M204V and L180M mutations in treatment-naïve patients (0.12% and 0.13%, respectively) were more than 100-fold lower than those (13.7% and 16.1%, respectively) in the treatment-failure patients (*p*<0.001). The frequencies of the V173L and M204I mutations were also significantly lower than those in the treatment-failure patients (*p*<0.001 and *p* = 0.01, respectively; Fig. 3B). Among the drug-resistance mutations that were specific to ETV, ADV, and LdT, only the N236T mutation was detected in one treatment-naïve patient. No drug-resistant mutations were detected in any of these treatment-naïve patients by population sequencing.

**Table 2:** Detection of drug-resistance mutations in treatment naive patients by PASS.

Patient	Genotype	VL (Log_10_ IU/ml)	HBeAg	No. of analyzed genomes	% of viruses with mutations	No. of mutations	No. of linkages
N047	C	8.0	+	2,772	0.44	4	1
N048	C	7.4	+	1,616	0.19	4	1
N049	B	7.9	+	1,010	0.20	3	1
N050	B	7.7	+	644	0.31	3	1
N051	C	7.7	+	327	0.62	4	1
N052	C	7.8	+	2,107	0.29	4	1
N053	D	7.6	+	1,120	0.27	2	0
N054	B	7.2	+	2,223	0.09	1	0
N055	C	7.2	+	317	0.00	0	0
N056	B	7.6	+	444	0.23	1	0
N057	C	6.9	+	2,352	0.38	1	0
N058	C	6.6	+	1,269	0.16	2	0
N059	C	7.7	+	1,179	0.00	0	0
N060	B	7.6	+	1,141	0.00	0	0
N061	C	6.0	+	826	0.24	1	0
N062	B	7.9	+	921	0.11	1	0
N063	C	5.8	+	1,303	3.15	4	5
N064	C	5.5	+	1,821	0.16	2	0
N065	C	8.2	+	1,159	0.35	1	0
N066	C	6.1	+	1,323	1.14	2	1
N067	C	7.7	+	638	0.32	2	0
N068	C	7.9	+	1,743	0.12	2	0
N069	C	7.7	+	1,302	0.08	1	0
N070	C	6.2	−	1,625	0.55	2	1
N071	C	7.0	+	449	3.79	3	2
N072	B	6.3	+	1,407	0.43	2	0
N073	C	5.5	+	1,386	0.29	1	0
N074	B	8.0	+	1,520	0.00	0	0
N075	C	7.9	+	881	0.00	0	0
Mean		7.2 (5.5–8.0)		1,257 (317–2,772)	0.32 (0.09–3.79)	1.8 (0–4)	1.3 (0–5)

**Figure 4:**
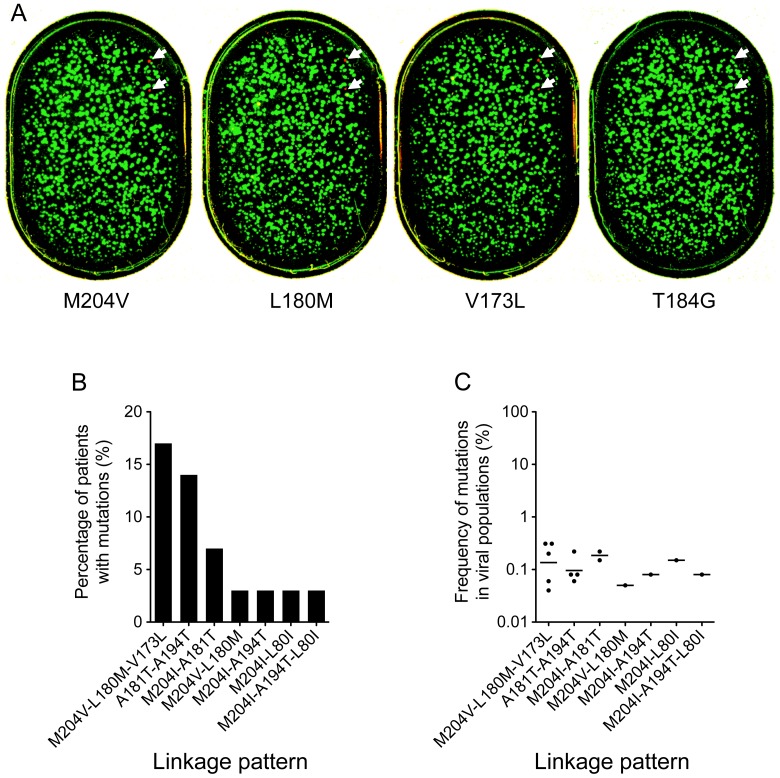
Linkage analysis of the drug-resistance mutations in the individual viral genomes of treatment-naïve patients. The immobilized 1010 PCR amplicons from patient N049 were sequentially probed in the same gel with 13 different sequencing primers (A). Drug-resistance mutations at M204V, L180M, and V173L sites were detected. No drug-resistance mutations were detected at the other 10 sites (L80V, L80I, A181V, A181T, T184G, A194T, S202I, M204I, N236T, and M250V), and one representative image (T184G) is shown. Each spot represents one individual wt (green) or mutant (red) base at the analyzed position. Two viruses with linked M204V, L180M, and V173L mutations are indicated by arrows. The percentages of patients with different linkage patterns (B) and the frequencies of each linkage pattern in the viral population (C) were determined. Each dot indicates the frequency of a linkage of mutations in the viral population in a patient. The geometric means of the frequencies of viruses with different linkage patterns in the viral populations are shown as bars.

Although the frequencies of the mutations in the viral populations of the treatment-naïve patients were very low (<2%; Fig. 3B), seven linkage patterns were detected in 10 patients (Fig. 4A and 4B). The majority of these linkage patterns (6 of 7) contained primary LAMr mutations (M204V or M204I). Importantly, three linkages (M204V-L180M, M204V-L180M-V173L, and M204I-L80I) were also the predominant drug-resistance mutations that were detected in the treatment-failure patients and were significantly more frequently detected than any of the other linkages (*p*<0.01). All linkage patterns were present at very low frequencies (0.04%–0.14%) in the viral populations (Fig. 4C). These results indicate that LAMr mutations, especially those linked mutations among which the secondary mutations can render drug-resistant viruses more resistant and compensate the fitness loss caused by M204V/I [12]–[16], are frequently present in the treatment-naïve patients and may play a critical role in the low genetic resistance barrier to LAM.

## Discussion

We characterized the drug-resistance mutations in both the treatment-failure and -naïve patient groups using the highly sensitive PASS assay to understand the mechanisms of drug resistance in HBV infection. We found that LAMr mutations were present in all of the treatment-failure patients who were treated with or without LAM, and two linkage patterns of LAMr mutations (M204V-L180M and M204V-L180M-V173L) predominated the drug-resistance viral populations. These results are in agreement with previous observations [12]–[16], [23]–[26] and further confirm that viruses with M204V-L180M and/or M204V-L180M-V173L linkage patterns have a major role in resistance to LAM *in vivo*. Importantly, LAMr mutations dominated minority drug-resistance mutations in the treatment-naïve patients. Moreover, the minority linked LAMr mutations (M204V-L180M, M204V-L180M-V173L, and M204I-L80I) that were the same as those predominant drug-resistance mutations in the treatment-failure patients were the major linkage patterns in the treatment-naïve patients. Our results strongly suggest that one major mechanism for CHB patients to develop resistance to LAM is due to the selection of preexisting linked minority drug-resistance mutations.

Previous studies have demonstrated that preexisting drug-resistance mutations have an important role in the development of drug resistance in treated AIDS patients [29]–[33]. It has been suspected that preexisting drug-resistance mutations have a similar role in drug resistance in treated CHB patients [14], [25], [34], [35]. However, this hypothesis has not been clearly supported because minority linked drug-resistance mutations present in less than 1% of the population could not be reliably detected [22], [26], [35]–[37]. Low frequency LAMr mutations have been detected in treatment-naïve patients by real-time PCR and UDPS methods [22], [26], [35], [37]–[39]. However, linked preexisting drug-resistance mutations have not been detected even when the highly sensitive UDPS method was used [26]. Thus, the detection of linked LAMr mutations present at very low frequencies in treatment-naïve patients using the PASS assay in this study strongly suggests that they play an important role in the development of drug resistance. This is supported by several lines of evidence. First, secondary/compensatory mutations (L180M, V173L, and L80I) always occurred together with the primary mutations (M204V and M204I) in the patients who failed the treatment due to drug resistance [7], [12]–[15], [25], [40]. Second, the strongest evidence comes from a study in which multiple clones from longitudinal samples collected bimonthly at baseline until virological breakthrough were analyzed in four patients [25]. The study showed that linked mutations (M204V-L180M and M204V-L180M-V173L) always appeared earlier than the individual M204V mutation and predominated the viral population during the entire virological breakthrough period in three patients [25]. Third, three linkage patterns of LAMr mutations (M204V-L180M, M204V-L180M-V173L, and M204I-L80I), which are the most frequently detected in treatment-failure patients [4]–[7], [12], [13], [15], [25], [26], [41], were all found in the treatment-naïve patients. Importantly, the viruses carrying linked primary and secondary mutations will not significantly lose their fitness and therefore can be frequently detected because the secondary mutations can compensate the fitness loss caused by the M204V/I mutations [12]–[16]. Thus, this finding strongly suggests that the most plausible explanation for the low genetic barrier to LAM is due to the selection of preexisting linked LAMr mutations instead of the sequential addition of secondary/compensatory mutations after the emergence of the primary M204V mutation [4], [7], [42].

The UDPS method has recently been used to study minority drug-resistance mutations in treatment-failure and -naïve patients [22], [26], [36]. This method can reliably detect minority drug-resistance mutations present at 0.1%–1% of the viral population, but the linked mutations cannot be assessed by this method due to the short read length [22], [26], [36]. Because the linked mutations were present at very low frequencies (0.04%–0.31%), these would not be detected by other assays. In our previous study, we analyzed all possible mismatches for all four bases by analyzing 1,899,000 sites and found 11 mismatches [19]. This yields an error rate of 6×10^−6^. Since extracted HBV DNA genomes were directly analyzed by PASS, we expected the limit for the detection of minority populations by PASS was the same as what we reported earlier (0.0006%), which is 67-fold lower than the lowest frequency (0.04%) that was detected in clinical samples in this study. Thus, the low frequency mutations that were detected by PASS represent the actual minority drug-resistance populations *in vivo*.

Drug-resistance mutations (T184G, M250V, N236T, and S202I) specific to three drugs (ADV, ETV, and LdT) that were used to treat CHB in this cohort were rare, and few linkages between these mutations and LAMr mutations were detected in the treatment-failure patients. The rare detection of individual or linked drug-resistance mutations in the treatment-naïve patients may explain why the development of drug resistance is less frequent and much slower in patients who are treated with ADV and ETV than with LAM. This finding indicates that the mechanisms of drug resistance for these drugs are different from those for LAM. One limitation of this study is the analysis of cross-sectional samples. Future prospective studies using baseline and longitudinal treatment-failure samples from the same individuals can further confirm our findings and discover new drug-resistance mechanisms for other NAs. Linked mutations in genes other than the RT gene may also play a role in selection of preexisting drug-resistance mutations or acquisition of insensitivity to NAs, even in absence of any drug-resistance mutations in the RT gene, during the development of drug resistance. Since those mutations were not in the gene fragment amplified in the PASS assay, they were not investigated in this study.

Cross resistance is common among NAs [6], [21]. However, this should not significantly affect our results from analysis of five major LAMr associated mutations (M204V, M204I, L180M, L80I and V173L). The M204I mutation is shared by LAM and LdT. It could be selected by LAM or LdT in treatment-failure patients since both were used to treat CHB patients in this study. Since the M204I mutation was detected at the lowest frequency (30%) among five mutations in treatment-failure patients (Fig. 1) and since it is not possible that the M204I mutation in all patients were due to LdT, it is unlikely that the portion of the M204I mutation selected by LdT could significantly affect our analysis. Resistance to ETV requires additional mutations in addition to the LAMr mutations. Thus, it was expected that when a patient failed ETV due to development of drug resistance, the LAMr mutations would be detected and would render LAM ineffective.

Our findings have important implications for the management of HBV-infected patients. Due to the low genetic barrier to LAM resistance, LAM is not recommended to be included in the first-line drugs or used as monotherapy to treat CHB patients in the United States [6], [42]. However, because LAM is inexpensive and well tolerated even in patients with decompensated cirrhosis, it was still recommended as the second-line therapy when more potent dugs with high genetic barriers to resistance are not available or appropriate in European and Asia-Pacific countries in the recent treatment guidelines [43]. The majority of the CHB patients (75%) live in the Asia Pacific region and one-third (∼130 millions) of the CHB patients reside in China [44], [45]. However, LAM, like other approved NAs, is still recommended as the first-line monotherapy to treat CHB patients in these regions in the recent treatment guidelines [20], [46]. Our findings strongly suggest that the use of LAM will not benefit treatment-failure or treatment-naïve patients. Firstly, the preexisting minority LAMr mutations can quickly lead to treatment failure due to drug resistance. Secondly, the development of LAMr mutations in all treatment-failure patients, irrespective of the inclusion of LAM in the regimen, will severely comprise the efficacy of LAM when used as a second-line drug. Finally, since patients can quickly develop drug resistance to ETV in the presence of LAMr mutations [4], the LAMr mutations can significantly compromise the efficacy of ETV. Moreover, our findings also indicate that minority drug-resistance mutations for LAM and other NAs should be determined prior to initiating or changing antiviral regimens to most effectively treat CHB patients by selecting only sensitive drugs.

## Supporting Information

Table S1 **Frequency of drug-resistance mutations detected in the viral populations in treatment failure patients by PASS.**
(DOCX)

Table S2 **Frequency of drug-resistance mutations detected in the viral populations in treatment naive patients by PASS.**
(DOCX)
