# Supplementary material for: Association of Preexisting Drug-Resistance Mutations and Treatment Failure in Hepatitis B Patients
Source: PLoS One. 2013 Jul 30;8(7):e67606. doi: 10.1371/journal.pone.0067606 (PMC3728369; doi:10.1371/journal.pone.0067606)
Supplement: Table S1 — Frequency of drug-resistance mutations detected in the viral populations in treatment failure patients by PASS. (DOCX) [file pone.0067606.s001.docx]

| Table S1. Frequency of drug-resistance mutations detected in the viral populations in treatment failure patients by PASS | | | | | | | | | | | | | |
| --- | --- | --- | --- | --- | --- | --- | --- | --- | --- | --- | --- | --- | --- |
| Patient | L80V | L80I | V173L | L180M | A181T | A181V | T184G | A194T | S202I | M204V | M204I | N236T | M250V |
| N001 | - | 1.69 | 1.95 | 1.95 | - | - | - | - | - | 1.95 | - | - | - |
| N002 | - | - | 29.26 | 29.26 | 3.19 | - | - | - | - | 33.91 | - | 0.40 | - |
| N003 | - | - | 12.74 | **97.45** | - | - | - | - | - | **97.77** | - | - | - |
| N004 | - | - | 45.94 | 45.94 | **3.75** | - | - | - | - | 45.31 | - | - | - |
| N005 | - | **84.24** | - | 0.49 | 0.49 | - | - | - | - | 0.49 | **97.04** | - | - |
| N006 | - | - | 12.00 | 19.33 | - | - | - | - | - | 16.00 | - | - | - |
| N007 | - | - | 5.59 | - | - | - | - | - | - | 6.18 | - | - | - |
| N008 | - | - | 1.38 | **99.56** | - | - | - | - | - | **99.56** | - | - | - |
| N009 | - | - | 2.34 | 6.22 | - | - | - | - | - | 6.52 | - | - | - |
| N010 | - | - | **34.78** | **42.24** | - | **22.98** | - | - | - | **39.75** | 4.97 | **20.5** | 14.29 |
| N011 | - | - | 9.86 | 14.02 | 0.92 | - | - | - | - | 14.02 | - | - | - |
| N012 | - | - | 55.2 | 46.45 | 0.63 | - | - | - | - | 61.42 | - | - | - |
| N013 | - | - | 14.61 | 17.78 | 0.20 | - | - | - | - | 18.48 | - | - | - |
| N014 | - | **99.61** | - | **97.69** | - | - | - | - | - | 0.84 | **99.61** | - | - |
| N015 | - | - | 9.06 | 14.09 | 6.49 | - | - | - | - | 17.11 | - | - | - |
| N016 | - | - | - | 0.34 | 8.17 | - | - | - | - | 2.20 | - | - | - |
| N017 | - | - | 3.42 | 8.22 | - | - | - | - | - | 6.85 | - | - | - |
| N018 | - | - | - | **93.58** | 0.21 | - | - | - | - | **93.58** | - | - | - |
| N019 | - | - | - | 1.71 | # | - | - | - | - | 16.35 | - | **78.33** | - |
| N020 | - | **67.12** | - | 0.30 | **5.68** | - | - | 0.30 | - | 0.37 | **66.82** | - | - |
| N021 | - | 0.38 | 43.98 | 51.00 | **15.91** | - | - | - | - | 52.26 | 0.63 | - | - |
| N022 | - | - | - | 0.15 | 0.46 | - | - | 0.31 | - | 0.31 | - | - | - |
| N023 | - | - | 6.44 | - | 0.42 | - | - | - | - | 8.32 | - | - | - |
| N024 | - | - | 4.13 | - | 0.83 | - | - | - | - | 4.25 | - | - | - |
| N025 | - | - | 5.35 | - | - | - | - | - | - | 15.51 | - | - | - |
| N026 | - | - | 21.47 | 22.98 | 0.68 | - | - | - | - | 28.95 | - | - | - |
| N027 | - | 66.82 | 84.79 | 80.18 | - | - | - | - | - | 85.25 | - | - | - |
| N028 | - | - | - | - | - | - | - | - | - | 8.86 | **89.71** | - | - |
| N029 | 19.49 | 0.96 | **49.20** | **23.00** | - | - | - | - | - | 14.38 | **44.41** | - | - |
| N030 | - | - | 9.37 | 9.73 | - | - | - | 0.09 | - | 12.47 | - | - | - |
| N031 | - | 14.15 | 0.64 | **91.32** | 0.32 | - | **89.39** | - | - | **91.32** | 15.11 | - | - |
| N032 | - | - | 4.36 | 19.63 | - | - | - | - | - | 20.25 | - | - | - |
| N033 | - | **63.07** | 18.33 | **96.50** | - | - | - | - | - | **26.15** | **73.85** | - | - |
| N034 | - | - | 11.17 | 12.73 | - | - | - | - | - | 11.32 | - | - | - |
| N035 | - | 3.85 | 12.61 | 24.15 | - | - | 5.77 | - | - | 25.21 | 2.99 | - | - |
| N036 | - | - | 9.30 | 12.36 | **77.20** | - | - | - | - | 12.36 | - | - | - |
| N037 | - | 0.52 | 12.76 | 13.41 | **48.05** | - | - | - | - | 13.41 | 1.43 | - | - |
| N038 | - | - | 42.60 | 42.60 | 0.37 | - | - | - | - | 42.60 | - | - | - |
| N039 | - | - | 9.81 | - | 0.40 | - | - | - | - | 9.81 | **82.93** | - | - |
| N040 | - | 16.32 | 15.48 | 17.57 | - | - | - | 1.67 | - | 16.32 | - | - | - |
| N041 | - | - | 83.90 | 81.29 | - | - | - | - | - | 87.53 | - | - | - |
| N042 | - | - | - | **88.56** | 1.06 | - | - | - | - | 8.05 | **91.53** | - | - |
| N043 | - | 9.95 | 9.00 | **98.10** | - | - | - | - | - | **91.94** | - | - | - |
| N044 | - | 0.35 | 4.80 | 6.14 | 0.23 | - | - | - | - | 5.67 | **93.86** | - | - |
| N045 | - | - | **26.32** | **90.79** | - | - | - | - | - | **90.79** | - | - | # |
| N046 | - | - | - | 1.63 | 0.28 | - | - | - | - | 1.49 | - | - | - |
| Mean | 19.49 | 7.65 (0.35-99.61) | 11.54 (0.64-84.79) | 16.09 (0.15-99.56) | 1.34 (0.20-77.20) | 22.98 | 22.71 (5.77-89.39) | 0.34 (0.09-1.67) |  | 13.70 (0.31-99.56) | 24.46 (0.63-99.61) | 8.63 (0.40-78.33) | 14.29 |
| Boldface: mutations detected by population sequencing | | | | | | |  |  |  |  |  |  |  |
| #: mutations positive by population sequencing only | | | | | |  |  |  |  |  |  |  |  |
