# Supplementary material for: Association of Preexisting Drug-Resistance Mutations and Treatment Failure in Hepatitis B Patients
Source: PLoS One. 2013 Jul 30;8(7):e67606. doi: 10.1371/journal.pone.0067606 (PMC3728369; doi:10.1371/journal.pone.0067606)
Supplement: Table S2 — Frequency of drug-resistance mutations detected in the viral populations in treatment naive patients by PASS. (DOCX) [file pone.0067606.s002.docx]

| Table S2. Frequency of drug-resistance mutations detected in the viral populations in treatment naive patients by PASS | | | | | | | | | | | | | |
| --- | --- | --- | --- | --- | --- | --- | --- | --- | --- | --- | --- | --- | --- |
| Patient | L80V | L80I | V173L | L180M | A181T | A181V | T184G | A194T | S202I | M204V | M204I | N236T | M250V |
| N047 | - | - | 0.07 | 0.04 | 0.36 | - | - | - | - | 0.04 | - | - | - |
| N048 | - | - | 0.06 | 0.06 | - | - | - | - | - | 0.06 | - | 0.12 | - |
| N049 | - | - | 0.20 | 0.20 | - | - | - | - | - | 0.20 | - | - | - |
| N050 | - | - | 0.31 | 0.31 | - | - | - | - | - | 0.31 | - | - | - |
| N051 | - | - | 0.31 | 0.31 | 0.31 | - | - | - | - | 0.31 | - | - | - |
| N052 | - | 0.05 | - | 0.09 | 0.19 | - | - | - | - | 0.05 | - | - | - |
| N053 | - | - | - | - | 0.09 | - | - | - | - | 0.18 | - | - | - |
| N054 | - | - | - | - | 0.09 | - | - | - | - | - | - | - | - |
| N055 | - | - | - | - | - | - | - | - | - | - | - | - | - |
| N056 | - | - | 0.23 | - | - | - | - | - | - | - | - | - | - |
| N057 | - | - | - | - | 0.38 | - | - | - | - | - | - | - | - |
| N058 | - | - | - | - | 0.08 | - | - | - | - | 0.08 | - | - | - |
| N059 | - | - | - | - | - | - | - | - | - | - | - | - | - |
| N060 | - | - | - | - | - | - | - | - | - | - | - | - | - |
| N061 | - | - | - | - | 0.24 | - | - | - | - | - | - | - | - |
| N062 | - | - | - | - | - | - | - | - | - | 0.11 | - | - | - |
| N063 | - | 0.31 | - | - | 1.61 | - | - | 0.31 | - | - | 1.46 | - | - |
| N064 | - | - | - | - | 0.11 | - | - | 0.05 | - | - | - | - | - |
| N065 | - | - | - | - | 0.35 | - | - | - | - | - | - | - | - |
| N066 | - | - | - | - | 1.06 | - | - | 0.15 | - | - | - | - | - |
| N067 | - | - | - | - | 0.16 | - | - | 0.16 | - | - | - | - | - |
| N068 | - | 0.06 | - | - | - | - | - | 0.06 | - | - | - | - | - |
| N069 | - | - | - | - | 0.08 | - | - | - | - | - | - | - | - |
| N070 | - | - | - | - | 0.49 | - | - | 0.12 | - | - | - | - | - |
| N071 | - | - | - | - | 1.11 | - | - | 1.11 | - | - | 2 | - | - |
| N072 | - | - | - | - | - | - | - | 0.36 | - | 0.07 | - | - | - |
| N073 | - | - | - | - | 0.29 | - | - | - | - | - | - | - | - |
| N074 | - | - | - | - | - | - | - | - | - | - | - | - | - |
| N075 | - | - | - | - | - | - | - | - | - | - | - | - | - |
| Mean |  | 0.10 (0.06-0.31) | 0.16 (0.06-0.31) | 0.13 (0.04-0.31) | 0.27 (0.08-1.61) |  |  | 0.18 (0.05-1.11) |  | 0.11 (0.04-0.31) | 1.71 (0.20-1.46) | 0.12 |  |
